# Social isolation and mental well-being among Korean older adults: a focus on living arrangements

**DOI:** 10.3389/fpubh.2024.1390459

**Published:** 2024-04-24

**Authors:** Geon Lee, Chulwoo Kim

**Affiliations:** ^1^Department of Public Administration, Hanyang University, Seoul, Republic of Korea; ^2^Department of Public Administration, Gachon University, Seongnam, Republic of Korea

**Keywords:** social isolation, loneliness, living arrangements, mental health, older adults

## Abstract

**Introduction:**

The aging population in South Korea, characterized by an increasing number of older adults living alone, has raised concerns about its implications on mental health, specifically social isolation and loneliness that accompanies solitary living arrangements. This study explores the impact of living arrangements on the mental well-being of Korean older adults by focusing on the prevalence of depression and the role of social isolation in the context of evolving family structures and the COVID-19 pandemic.

**Methods:**

This cross-sectional study analyzed the responses of older adults aged 65 years and above (mean: 73.1, SD: 5.1) by using data from the Korea National Health and Nutrition Examination Survey conducted in 2018 and 2020. In total, responses from 3,365 older adults (1,653 in 2018 and 1,712 in 2020) were employed in this research. The participants’ mental health status was assessed using the Patient Health Questionnaire-9, with living arrangements categorized by household size. A zero-inflated Poisson regression analysis was employed to investigate the relationship between living arrangements and depression severity, controlling for demographic, socioeconomic, and psychological factors.

**Results:**

The study found that older adults living with others exhibited a lower depression severity than those living alone. Notably, the severity of depression decreased as the number of household members increased up to a certain threshold. Socio-economic factors, such as income level, marital status, and psychological stress were also identified as significant predictors of depression severity. However, the COVID-19 pandemic did not have a statistically significant impact on depression rates among older adults during the study period.

**Conclusion:**

Living arrangements play a critical role in the mental health of Korean older adults, with solitary living being associated with higher levels of depression. These findings underscore the importance of social support systems and suggest the need for policies and interventions that promote social connectivity and address the challenges of loneliness faced by them. Future research should explore longitudinal and qualitative studies to further understand causal relationships and develop targeted interventions to improve the mental well-being of the aging population.

## 1 Introduction

Global increases in life expectancy has resulted in a growing population of older adults, coinciding with evolving family structures that have contributed to smaller family units (1). Consequently, older adults, particularly in industrialized nations, are increasingly residing alone (2–5). The *per capita* increase in the prevalence of the older adults living alone stems from a combination of ongoing social transformations and the aging demographic (6). This phenomenon has led to negative subjective well-being among older adults (7). Older adults living alone are at an increased risk of experiencing social isolation and loneliness (8). With diminishing family connections, accessing social and emotional support has become more challenging, leading to potential mental health issues (7). Furthermore, cultural shifts and advancements in industrialization and urbanization have disrupted traditional family based support frameworks (9).

Over the years, the landscape of living arrangements in South Korea has witnessed significant transformation (1), with profound implications for the mental health of the country’s aging population. This study examined the impact of these evolving living arrangements on the mental health of older adults, particularly in the context of the Coronavirus (COVID-19) pandemic. With a notable increase in single-person households, especially among older adults, South Korea presents a unique case for understanding the intricate relationship between living conditions and mental health in older adults (10).

Before the pandemic, South Korea had witnessed a surge in the number of single-person households, a trend that extended to older adults. This shift, emblematic of broader societal changes, has raised concerns about the phenomenon of “lonely deaths,” in which older adults living alone pass away unnoticed, a stark manifestation of social isolation (11, 12). Additionally, the poverty rate among older adults in Korea is one of the highest among the OECD countries, compounding the challenges faced by this demographic (13, 14).

According to official figures from Statistics Korea, older adults comprise 15.9% of the total population, and the proportion of older adults living alone among the older population in South Korea is approximately 20% as of 2020 (15). The increasing number of older adults living alone has led to several social problems. Lonely deaths among older adults is increasing due to an increasing number of them living alone (11, 16). The proportion of older adults over 60 among all lonely deaths has increased over the past 5 years to 37.1% in 2017, 42.7% in 2019, and 47.5% in 2021 (17). In addition to lonely deaths, suicide rates among older adults are increasing. As of 2020, the suicide rate among older adults in South Korea was higher than in other age groups, with an approximate rate of 30 suicides per 100,000 between the ages of 40 and 60, but 38.8 in the 70s and 62.6 in the 80s age group, demonstrating an increase of suicidal rate with age (18). In particular, the average suicide rate among older adults in South Korea was 46.6 suicides per 100,000, more than double the OECD average of 17.2, and the highest among OECD countries (18). The suicide rate among older adults was found to be associated with living arrangements, showing that the prevalence of suicidal ideation among older adults living alone is more than twice as compared with those living with others (19).

The COVID-19 pandemic period represented a highly atypical phase, characterized by enforced isolation and heightened stress levels, challenging the normative conditions of social interaction and mental well-being (20–22). Specifically, the need to measure the impact of this period arises from its unprecedented global disruption, which has significantly altered daily life, especially for vulnerable populations such as older adults.

South Korea is an exception in this context. Despite the global struggle, South Korea’s effective management and containment strategies for COVID-19 have been widely recognized, potentially mitigating the severity of isolation impacts compared with other countries (23, 24). This unique scenario makes South Korea an ideal case study for examining the effects of the pandemic on the mental health of older adults in a managed environment.

Understanding these impacts in the South Korean context not only provides insights into the resilience and vulnerabilities of older adults during such crises, but also offers a nuanced understanding of how well-defended communities navigate the challenges posed by global pandemics. This analysis is crucial for tailoring interventions and policies that support the mental health of older adults, not just in times of global health crises, but in any situation that isolates them from their social networks and support systems.

This study explores the nuanced ways in which living arrangements affect the mental health of Korean older adults, both before and in the wake of the COVID-19 pandemic. By examining variables such as social support systems and socio-economic challenges unique to this demographic, this study aims to shed light on the critical issue of mental health among Korean older adults and suggests pathways for policy and community interventions.

## 2 Materials and methods

### 2.1 Data source

The datasets used in this study was from the Korea National Health and Nutrition Examination Survey (KNHANES), conducted by the Korea Centers for Disease Control and Prevention, an agency of the South Korean government. The survey participants were chosen through a comprehensive process involving questionnaire surveys and medical examinations. While the number of respondents may exhibit slight variations annually, the 2018 KNHANES employed in this study involved 13,000 individuals across 4,416 households. In the 2020 KNHANES, 14,000 individuals from 4,800 households were surveyed. The survey encompassed individuals aged 1 year and above, covering a broad age range of up to 100 years. The participants were categorized into children, adolescents, and adults. Each group underwent tailored survey inquiries based on their individual characteristics. In the case of adolescents, parents responded on their children’s behalf. The survey content was organized into two modules, with one module consistently applied annually and the other subject to periodic replacement.

The sampling approach used in this study involved area probability sampling with multi-cluster sampling. Under this sampling design, once the survey area was determined, households were chosen through household member verification, facilitated by interviewers. The interviewer, in collaboration with local health and community centers, examined the survey area boundaries and determined the appropriate number of households. In accordance with the designated household count in the survey area, the interviewers further divided the area into zones and selected 25 households from each designated zone. Subsequently, the chosen households underwent a visitation process to confirm essential survey information and collect contact details for scheduling future phone interviews for participation in the survey. Selected households were notified of their participation through the “Notice of Household Selection for the Korea National Health and Nutrition Examination Survey.” This notice, issued by the head of the local government, was distributed 1 month before the survey commenced. A week before the survey initiation, the interviewers proactively reached out to selected households to coordinate a preliminary appointment. This early engagement aimed to facilitate communication between the respondent and interviewer, allowing for a pre-determined mutual arrangement of interview times. Interviewers conducted the interviews using the Computer-Assisted Personal Interviewing (CAPI) method.

### 2.2 Measures

#### 2.2.1 Mental health

Various tools exist to assess an individual’s mental health, and the Patient Health Questionnaire-9 (PHQ-9) is a commonly employed instrument to gauge mental well-being, including depressive symptoms (25, 26). The PHQ-9, a nine-item scale crafted by Spitzer, Kroenke and Williams evaluates mental health conditions. The PHQ-9 scale includes the following question: “Over the last 2 weeks, how often have you been bothered by any of the following problems?” The items listed are: (1) Little interest or pleasure in doing things, (2) Feeling down, depressed, or hopeless, (3) Trouble falling or staying asleep, or sleeping too much, (4) Feeling tired or having little energy, (5) Poor appetite or overeating, (6) Feeling bad about yourself—or that you are a failure or have let yourself or your family down, (7) Trouble concentrating on things, such as reading the newspaper or watching television, (8) Moving or speaking so slowly that other people could have noticed? Or the opposite—being so fidgety or restless that you have been moving around a lot more than usual, and (9) Thoughts that you would be better off dead or of hurting yourself in some way. Each item is rated on a four-point scale, ranging from 0 (“Not at all”) to 3 (“Nearly every day”).

In this study, we used the Korean-translated version of the PHQ-9 scale, as used in the KNHNES, to assess mental health. The original PHQ-9 and its Korean translation have demonstrated validity and reliability within the medical community (27). In this study, the Cronbach’s alpha coefficient for the scale was 0.80 for both samples, attesting to its reliability. Furthermore, we conducted confirmatory factor analysis (CFA) to ascertain the scale’s validity, revealing significant findings ( $\chi^{2}$ =858.12, *p* < 0.001; CFI = 0.97; TLI = 0.95; RMSEA = 0.06; SRMR = 0.03). Following prior studies, we consolidated the nine items into a single variable in the statistical models owing to their adequate reliability and validity.

#### 2.2.2 Living arrangement

Living arrangement is the main independent variables affecting the mental health of older adults. This was measured by the number of household members. The response category for this survey item ranged from single to eight or more. As households with five or more people are relatively rare, this study recoded four household categories ranging from single to four or more people, creating dummy variables in the statistical models.

#### 2.2.3 Covariate

Statistical models using nonexperimental observational data are indispensable to account for variables that can impact the outcome variable. In this study, we incorporated an array of covariates into the statistical models to account for their potential influences on the mental health of older adults. Demographic, socioeconomic, and subjective stress variables were used as covariates in the statistical models. Regarding demographic variables, gender served as a fundamental control variable, as men and women may experience varying levels of mental health. Age is another crucial control variable, as we anticipated variations in mental health conditions based on age, despite focusing on older adults aged 65 years and above. For socioeconomic variables, marital status was treated as a dichotomous variable: married or unmarried. Household income was also considered an important control variable and was categorized into five brackets ranging from 1 (lowest) to 5 (highest). Education level was measured on a four-point scale ranging from less than 6 years (indicating no formal education) to graduate level (+13). Current employment status was dichotomously measured as “employed” or “unemployed.” Household ownership was categorized as either owner or non-owner. As for the current psychological status, which potentially influences mental health, we controlled for subjective stress levels, which ranged from 1(almost never) to 4(feels very stressed). Finally, a year variable was added to account for the impact of COVID-19, with 2018 representing the period before and 2020 representing the period during COVID-19.

### 2.3 Statistical analyses

To conduct our analysis, we focused on a specific subset of our data consisting of older adults aged 65 years and above. This subset was extracted from a larger dataset, which enabled us to concentrate on this demographic group. We used a statistical technique known as Zero-inflated Poisson Regression Analysis to investigate the factors influencing the mental health of older adults. Poisson regression models are commonly used when analyzing count data, where the outcomes are represented by nonnegative integer values. In our case, we were interested in understanding mental health outcomes, which often involve counts of symptoms or indicators. However, standard Poisson regression may not adequately handle datasets with an excess of zero counts, which is common in mental health studies where many individuals may not exhibit any symptoms. To address this issue, we employed a specialized form of Poisson regression, known as the Zero-inflated Poisson Regression. This technique was specifically designed to handle datasets with excess zero counts such as those encountered in our study.

**Figure 1:**
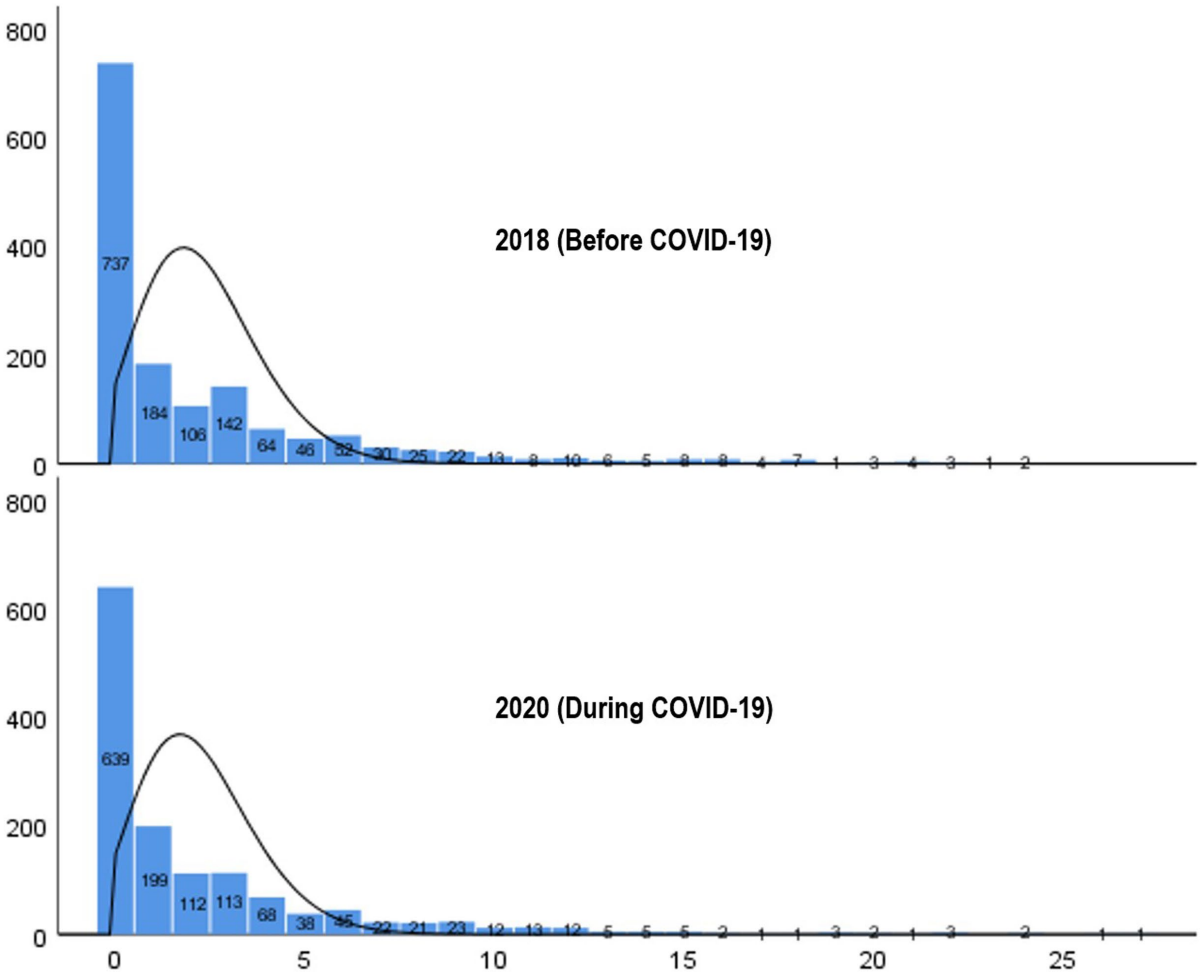
Distribution of scores of sums of PHQ-9 scales in 2018 and 2020.

As shown in Figure 1, the distribution of mental health scores (measured using the PHQ-9) for respondents in 2018 and 2020 was not normal. Instead, it exhibited a highly skewed distribution with a preponderance of values clustered toward the lower end, resembling a Poisson distribution with excess zeros. This phenomenon is indicative of overdispersion, in which the variance of the data exceeds what would be expected under a standard Poisson distribution. We evaluated two possible models: the standard Poisson model and the Zero-inflated Poisson (ZIP) model. To determine the most suitable model, we conducted the Vuong test, which is utilized for comparing non-nested models (28, 29). Based on the Z-statistics (0.15, 0.01) from the Vuong test for models incorporating only main effects and those including both main and interaction effects, we were unable to reject the null hypothesis at the level of 0.05 that the standard Poisson model and the ZIP model fits the data equally well. Despite the possibility of choosing either model, we ultimately selected the ZIP model due to additional criteria such as the Akaike Information Criterion (AIC) and the Bayesian Information Criterion (BIC). The AIC and BIC indices were lower for the ZIP model compared to the standard Poisson model, indicating a better fit (see Supplementary Appendix). All statistical analyses were conducted using the SAS 9.4 software.

## 3 Results

### 3.1 Sample characteristics

Table 1 outlines the sample characteristics of the two-time-point data used in our study. In the 2018 dataset, females comprise 57.65% of the sample, whereas males represent 42.35%. The age distribution indicates a concentration in the 65–74 age range, with 30.55% aged 65–69 and 50.51% aged 70–74. Households predominantly consist of two members (51.02%), followed by single-member households (23.81%). Marital status indicates a vast majority (99.09%) of married individuals. Educational attainment varies, with a significant proportion having no formal education (56.84%), while employment status indicates that 66.57% are unemployed. Income distribution skews toward lower categories, with 39.87% falling into the lowest-income bracket. For home ownership, the majority (73.71%) own their homes. Although there is a slight variation, the distribution of the 2019 sample conforms to this pattern.

**Table 1:** Sample characteristics.

	A: 2018 sample (before COVID-19)		B: 2020 sample (during COVID-19)		B - A (difference in percent)
	N	Percent (%)	N	Percent (%)	
**Gender**					
Male	700	42.35	739	43.17	0.82
Female	953	57.65	973	56.83	−0.82
Age					
65–69	505	30.55	500	31.81	1.26
70–74	447	30.51	502	31.93	1.42
75–79	202	13.79	221	14.06	0.27
80 +	311	21.23	349	22.20	0.97
**Number of household members**					
One	386	23.81	421	24.99	1.18
Two	827	51.02	902	53.53	2.51
Three	241	14.87	239	14.18	−0.69
Four +	167	10.30	123	7.30	−3.00
**Marital status**					
Married	1,638	99.09	1,702	99.47	0.38
Not married	15	0.91	9	0.53	−0.38
**Education**					
No formal education	864	56.84	704	51.24	−5.60
6 years	245	16.12	243	17.69	1.57
7–12 years	263	17.30	278	20.23	2.93
13 years +	148	9.74	149	10.84	1.10
**Employment status**					
Employed	523	34.43	512	37.26	2.83
Unemployed	996	65.57	862	62.74	−2.83
**Household income category**					
1 (lowest)	655	39.87	594	35.06	−4.80
2 (low)	432	26.29	504	29.75	3.46
3 (middle)	251	15.28	262	15.47	0.19
4 (high)	173	10.53	210	12.40	1.87
5 (highest)	132	8.03	124	7.32	−0.71
**House ownership**					
Yes	1,217	73.71	1,263	73.86	0.15
No	434	26.29	447	26.14	−0.15
Total	1,653	100.00	1,712	100.00	

### 3.2 Mental health disparity across factors

Table 2 shows the disparities in mental health, as measured by the PHQ-9 scale instrument, across various demographic and socio-economic factors. Gender exhibits a significant difference, with females reporting a higher mean score (2.73), compared to males (1.68), indicating potentially poorer mental health among females (*p* < 0.001). Age does not show a consistent pattern, with slight variations across age categories, but these are not statistically significant differences. The number of household members presents a notable disparity in mental health scores. Individuals living in households with a single-member report the highest mean score (3.07), indicating that potentially poorer mental health compared to older adults in households with two or more members (*p* < 0.001). Older adults who are not married report significantly higher mean scores (4.62) compared to married ones (2.25), suggesting that marital status may influence mental health outcomes. Similarly, those with no formal education report higher mean score (2.70) compared to those with higher levels of education (*p* < 0.001).

**Table 2:** Mental health (PHQ-9) disparity across factors.

Factor	Mean	Mean ± SD	F-statistic	*p*-value
**Gender**				
Male	1.68	1.68 ± 3.25	55.89	***
Female	2.73	2.73 ± 4.05		
**Age**				
65–69	2.23	2.23 ± 3.80	2.49	
70–74	2.07	2.07 ± 3.44		
75–79	2.65	2.65 ± 4.09		
80 +	2.50	2.50 ± 4.08		
**Number of household members**				
One	3.07	3.07 ± 4.48	13.51	***
Two	2.03	2.03 ± 3.42		
Three	1.99	1.99 ± 3.55		
Four +	2.06	2.06 ± 3.59		
**Marital status**				
Married	2.25	2.25 ± 3.71	8.28	**
Not married	4.62	4.62 ± 7.73		
**Education**				
No formal education	2.70	2.70 ± 4.18	18.82	***
6 years	2.14	2.14 ± 3.44		
7–12 years	1.82	1.82 ± 3.16		
13 years +	1.08	1.08 ± 2.30		
**Employment status**				
Employed	1.87	1.87 ± 3.29	18.72	***
Unemployed	2.50	2.50 ± 3.98		
**Household income category**				
1(lowest)	2.95	2.95 ± 4.45	15.35	***
2 (low)	2.13	2.13 ± 3.38		
3(middle)	1.81	1.81 ± 3.17		
4 (high)	1.65	1.65 ± 3.18		
5(highest)	1.52	1.52 ± 2.81		
**House ownership**				
Yes	2.04	2.04 ± 3.41	31.59	***
No	2.93	2.93 ± 4.52		
Year*				
2018 (before COVID-19)	2.32	2.32 ± 3.86	0.50	
2020 (during COVID-19)	2.22	2.22 ± 3.64		

*The average score of PHQ-9 for each year; **p < 0.01; ***p < 0.001.

Furthermore, employment status and household income category also demonstrate significant differences, with employed individuals and those in higher income categories reporting lower mean scores (*p* < 0.001) as compared with those who are unemployed and belong to lower income categories. House ownership is also associated with mental health disparities, with homeowners reporting lower mean scores (2.04) than non-homeowners (2.93). Finally, the year factor indicates no significant difference in mental health scores between 2018 (before COVID-19) and 2020 (during COVID-19), suggesting that while the pandemic may have influenced overall mental health trends, it did not manifest a significant difference within this specific age population. The proportion of the low-educated females living alone without ownership was found to be 7.8 percent in 2018 and 5.5 percent in 2020.

### 3.3 Effects of living arrangements and covariates on mental health

Table 3 presents the results of the Zero-inflated Poisson Regression analysis that examined the association between various factors and depression severity. Model 1 demonstrates that older adults living in larger households exhibited lower rates of depression than those living alone. Specifically, older adults residing in two-person households have an incidence rate ratio (IRR) of 0.85 (95% CI: 0.75–0.90), indicating a 15% reduction in depression severity. Similarly, older adults in three-person households show a greater reduction (IRR: 0.81; 95% CI: 0.73–0.89), while those in households of four or more persons have a modest reduction (IRR: 0.87; 95% CI: 0.78–0.90).

**Table 3:** Predictors of depression severity: incidence rate ratios from a Poisson regression model.

Variable	Model 1		Model 2	
	Depression severity		Depression severity	
	IRR (95% CI)	*p*-value	IRR (95% CI)	*p*-value
**Living arrangement**				
Single household (=reference)	1		1	
Two-person household	0.85 (0.75, 0.90)	***	0.81 (0.75, 0.89)	***
Three-person household	0.81 (0.73, 0.89)	***	0.74 (0.65, 0.85)	***
Four or more person household	0.87 (0.78, 0.90)	*	0.87 (0.76, 0.99)	*
**Year**				
2018 (=reference)	1		1	
2020 (COVID-19)	0.98 (0.93, 1.04)		0.91 (0.76, 1.09)	
**Household × Year**				
Single × COVID-19			1.01 (0.82, 1.25)	
Two-person × COVID-19			1.10 (0.90, 1.34)	
Three-person × COVID-19			1.22 (0.96, 1.56)	
**Gender**				
Male (=reference)	1		1	
female	1.06 (1.01, 1.13)	*	1.06 (1.01, 1.13)	*
**Age**				
65–69 (=reference)	1		1	
70–74	0.99 (0.92, 1.06)		0.99 (0.93, 1.06)	
75–79	1.08 (0.98, 1.16)		1.09 (0.98, 1.18)	
80 or more	1.07 (0.99, 1.17)		1.07 (0.87, 1.16)	
**Marital status**				
Married (=reference)	1		1	
Not-married	1.14 (0.88, 1.47)		1.12 (0.69, 1.45)	
**Household income category**				
3 (middle = reference)	1		1	
1 (lowest)	1.11 (1.02, 1.22)	*	1.11 (1.02, 1.22)	*
2 (low)	0.96 (0.88, 1.06)		0.96 (0.87, 1.05)	
4 (high)	0.99 (0.78, 1.11)		0.98 (0.87, 1.11)	
5 (highest)	0.92 (0.80, 1.06)		0.90 (0.78, 1.04)	
**Education**				
No formal education (=reference)	1		1	
6 years	0.93 (0.86, 1.00)		0.93 (0.86, 1.01)	
7–12 years	0.92 (0.84, 1.00)	*	0.91 (0.84, 0.99)	*
13 years +	0.65 (0.57, 0.75)	***	0.65 (0.57, 0.75)	***
**Employment status**				
Unemployed (=reference)	1		1	
Employed	0.86 (0.82, 0.92)	***	0.86 (0.81, 0.92)	***
**House ownership**				
No (=reference)	1		1	
Yes	0.87 (0.82, 0.92)	***	0.87 (0.82, 0.92)	***
**Subjective stress status**				
Almost never (=reference)	1		1	
Feels a little	1.11 (1.02, 1.20)	*	1.11 (1.02, 1.20)	*
Feels stressed	1.86 (1.71, 2.03)	***	1.86 (1.71, 2.03)	***
Feels very stressed	2.67 (2.40, 2.96)	***	2.65 (2.38, 2.94)	***

*p < 0.05; ***p < 0.001.

We hypothesized that the level of depression severity among older adults would be higher in the era of COVID-19 than the period before it; however, our study did not find a significant change in depression severity in the year 2020, marked by the COVID-19 pandemic, compared to the reference year of 2018 (IRR: 0.98; 95% CI: 0.93–1.04), suggesting that the pandemic’s onset did not have a uniform impact on depression rates across the study population.

For gender, females exhibit a slightly higher depression severity (IRR: 1.06; 95% CI: 1.01–1.13) compared to males. Participants aged 75–79 show a slight increase in depression severity (IRR: 1.08; 95% CI: 0.98–1.16), but it did not show statistical significance. Other age groups did not show significant differences from the 65–69 reference group. For income levels, compared to the middle income group, the lowest income group is more likely to experience depression severity (IRR: 1.11; 95% CI: 1.02. 1.22). Married individuals and those with higher household income, higher levels of education, employment, and house ownership are associated with lower depression severity, highlighting the protective effects of socio-economic stability and social support.

Notably, older adults reporting higher levels of stress reveal significantly increased depression severity, with those feeling very stressed showing nearly three times the severity (IRR: 2.67; 95% CI: 2.40–2.96) compared to those who almost never feel stressed.

To test our hypothesis that older adults living alone are more likely to experience increased depression severity during the pandemic compared to those living with family members, we included the year as a moderating variable in Model 2. Model 2 reveals that the three interaction effects of year and living arrangement on mental illness is not statistically significant. This suggests that the impact of living arrangements on mental health remains consistent, regardless of the COVID-19 period.

One of the strengths of ZIP model is that it allows us to analyze the likelihood of having excess zeros (or a PHQ-9 score of zero) for depressive symptom. Although this analytical property is not our main interest, we have additionally provided these analytical results. Table 4 presents the results of a logistic regression model from the ZIP model, focusing on the probability of an excess zero in PHQ-9 depression scores. The analysis indicates that all living arrangement categories, compared to the single household reference group, do not have significant effects on the likelihood of an excess of zero. Gender is found to be a significant predictor, with females showing lower log-odds of an excess zero outcome compare to males (B: −0.39, *p* < 0.001). Advancing age is associated with a higher likelihood of an excess zero, particularity evident in the 70–74 ae bracket (B:0.29, *p* < 0.01). Socioeconomic factors such as lower household income categories exhibit lower log-odds of an excess zero (B:-0.30 for the lowest income and − 0.36 for low income, *p* < 0.05), suggesting a possible greater acknowledgment of depressive symptoms among economically disadvantaged groups. Higher education beyond 13 years also correlates with a higher likelihood of an excess zero (B: 0.35, *p* < 0.05), as does being employed (B: 0.25, *p* < 0.01). Stress levels markedly affect the likelihood, with those feeling stressed or very stressed showing significantly lower log-odds of an excess zero (B: −1.78 and − 1.5, *p* < 0.001), emphasizing the influence of perceived stress on the non-reporting of depressive symptoms. These results consistent in Model 2, which contained the interaction effect, indicating that the COVID-19 variable had no effect.

**Table 4:** Zero-inflated logistic regression model: predicting non-depressive responses in PHQ-9.

Variable	Model 1		Model 2	
	Zero of PHQ-9		Zero of PHQ-9	
	Inflate estimate (95% CI)	*p*-value	Inflate estimate (95% CI)	*p*-value
**Living arrangement**				
Single household (=reference)				
Two-person household	0.11 (−0.11, 0.34)		−0.02 (−0.33, 0.29)	
Three-person household	0.21 (−0.11, 0.53)		0.03 (−0.40, 0.46)	
Four or more person household	0.18 (−0.17, 0.53)		0.18 (−0.25, 0.62)	
Year				
2018 (=reference)				
2020 (COVID-19)	−0.07 (−0.24, 0.10)		−0.31 (−0.87, 0.24)	
Household × Year				
Single × COVID-19			0.06 (−0.59, 0.72)	
Two-person × COVID-19			0.33 (−0.27, 0.94)	
Three-person × COVID-19			0.43 (−0.29, 1.16)	
**Gender**				
Male (=reference)				
female	−0.39 (−0.58, −0.20)	***	−0.40 (−0.58, 0.21)	***
**Age**				
65–69 (=reference)				
70–74	0.29 (0.07, 0.50)	**	0.29 (0.07, 0.51)	**
75–79	0.14 (−0.14, 0.43)		0.14 (−0.14, 0.43)	
80 or more	0.20 (−0.06, 0.47)		0.21 (−0.06, 0.48)	
Marital status				
Married (=reference)				
Not-married	1.14 (0.88, 1.47)		0.06 (−1.09, 11.13)	
Household income category				
**3 (middle = reference)**				
1 (lowest)	−0.30 (−0.58, −0.02)	*	−0.31 (−0.59, −0.03)	*		Model 1	Model 2	Variable	Zero of PHQ-9	Zero of PHQ-9		Inflate estimate (95% CI)	*p*-value	Inflate estimate (95% CI)	*p*-value
2 (low)	−0.36 (−0.63, −0.08)	**	−0.36 (−0.63, −0.09)	**
4 (high)	0.01 (−0.31, 0.34)		0.01 (−0.32, 0.33)	
5 (highest)	−0.32 (−0.70, 0.06)		−0.34 (−0.73, 0.04)	
**Education**				
No formal education (=reference)	1		1	
6 years	0.13 (−0.11, 0.37)		0.13 (−0.11, 0.38)	
7–12 years	0.06 (−0.18, 0.32)		0.05 (−0.19, 0.31)	
13 years +	0.35 (0.01, 0.68)	*	0.34 (0.01, 0.68)	*
**Employment status**				
Unemployed (=reference)				
Employed	0.25 (0.06, 0.45)	**	0.25 (0.06, 0.43)	**
**House ownership**				
No (=reference)				
Yes	0.02 (−0.18, 0.23)		0.03 (−0.18, 0.23)	
**Subjective stress status**				
Almost never (=reference)				
Feels a little	−0.93 (−1.13, 0.73)	***	−0.93 (−1.13, −0.73)	***
Feels stressed	−1.78 (−2.08, −1.48)	***	−1.79 (−2.09, −1.49)	***
Feels very stressed	−1.50 (−1.96, −1.03)	***	−1.50 (−1.97, −1.03)	***

*p < 0.05; **p < 0.01; ***p < 0.001.

## 4 Discussion and implication

### 4.1 Discussion

Our study investigated the impact of living arrangements on the mental health of older adults, with a particular focus on the severity of depression among older adults in South Korea. We found compelling evidence that older adults living with others experienced lower depression severity than those living alone. Furthermore, we identified a notable trend wherein the incidence of depression severity decreased as the number of household members increased: older adults living with one person fare better than those living alone, those with two family members fare better than those with one, and those with three or more members did not exhibit further improvement compared with those with two or more members. The result indicates several factors inherent in living with others, such as increased social interaction, emotional support, and a sense of belonging collectively act to buffer against the negative mental health impacts of loneliness and social isolation. These findings underscore the significance of living arrangements in influencing the mental health of older adults, which aligns with prior studies conducted across diverse national contexts (7, 30, 31).

From a theoretical standpoint, this study posits that the relationship between household environment and mental health is mediated by loneliness stemming from social isolation. While existing studies have predominantly focused on the impact of loneliness on mental health outcomes such as depression (32–34), our study offers a novel perspective by examining the household environment as a precursor to loneliness, which affects the mental well-being of older adults (35–37). Recent studies highlights the role of social support from household members in moderating the effects of loneliness on depression. These findings suggest that the nature of household dynamics directly correlates with the mental well-being of older adults, emphasizing the necessity of a supportive home environment (38, 39). We hypothesized that loneliness is not an inherent characteristic of older adults, but rather a consequence of their environment, with the household context playing a pivotal role. Older adults living alone are susceptible to social isolation and receive limited social and emotional support compared with those residing with family members, which can exacerbate feelings of loneliness and contribute to mental health decline (7).

Older adults who reside alone often experience compromised physical health, primarily due to limited access to healthcare without assistance. However, the correlation between older adults living alone and the experience of loneliness is a pressing concern, which subsequently contributes to a decline in their mental well-being (40, 41). Research indicates that loneliness primarily stems from social isolation characterized by a lack of meaningful social relationships (42). Consequently, the solitary living arrangements of older adults can foster an environment of social isolation, exacerbating feelings of loneliness, and thereby negatively impacting mental health. Studies have consistently identified loneliness as a significant factor that contributes to depressive symptoms in older adults (43, 44). The absence of daily social interactions and the lack of emotional and practical support mechanisms within the household can lead to heightened feelings of loneliness, thereby increasing the risk of depressive symptoms. The increased contact frequency with family and frequent participation in social activities, such as attending adult daycare centers, were associated with reduced loneliness among older adults (45). The importance of family social support in reducing loneliness levels among the older adult suggests that interventions designed to enhance social support systems could help mitigate loneliness and its detrimental effects on mental health (46).

While living alone has become increasingly prevalent in contemporary society, it is imperative to recognize the substantial economic costs associated with loneliness resulting from social isolation in many countries (47). Moreover, mental illness not only incurs an economic burden, but also poses significant social costs, including severe outcomes such as suicide, which warrant serious attention. It is essential to acknowledge that, while living alone may offer convenience and independence to younger generations, it represents a potentially hazardous social environment for older adults, posing risks to their mental and physical health.

### 4.2 Implications

South Korea is a high-risk society, with a high number of suicides among older adults. Given that the relationship between mental illnesses such as depressive symptoms and suicide (or suicidal ideation) is straightforward (48), depression due to loneliness may be one of the leading causes of suicide among older adults in South Korea. Our findings have several policy implications.

First, there is a pressing need to revamp the support programs for older adults living alone. Currently, South Korea offers various services for solitary older adults, such as safety verification, life skills education, and in-home assistance (49). However, this study indicates that older adults living with others tend to experience lower levels of depression. This underscores the importance of promoting co-living arrangements through policies and programs tailored to facilitate shared living among older adults, thereby mitigating loneliness and enhancing their mental well-being. Given the increased vulnerability of older adults living alone to social isolation and loneliness, it is imperative that these initiatives target this demographic. Additionally, housing policies should be re-evaluated to prioritize social connectedness and overall well-being, potentially exploring alternative housing options such as co-housing communities or age-friendly developments.

For example, the Naturally Occurring Retirement Community (NORC) program serves as a relevant model (50–53). Furthermore, NORCs residential communities for older adults fosters companionship and support among residents along with opportunities for collective activities and collaboration on common goals. Implementing similar collective residential programs for older adults living alone could effectively reduce social isolation and mitigate the mental health outcomes stemming from loneliness.

Second, our observation of reduced depression severity with increased household size up to a certain threshold underscores the potential benefits of larger households. Living with family members or others offers older adults both companionship and essential practical and emotional support, including assistance with health care needs which can be challenging for those living alone. This support system within the household helps alleviate loneliness and can significantly lessen the severity of depression (38, 39, 45). Policies encouraging multigenerational living arrangements or support systems for older adults cohabiting with family members can contribute to improved mental health outcomes. South Korea is typically categorized as a collectivistic culture (54). The country has changed from a large family system with multiple generations living together centered on the patriarch to a nuclear family system with increased individualism, leading to an increase in single-person households. It is important to emphasize the importance of family support and care for older adults.

Finally, it is crucial to acknowledge the socio-economic costs associated with loneliness and mental illness among older adults. Policies addressing social isolation not only enhance individual well-being but also alleviate economic burdens and prevent severe outcomes such as suicide. Given the correlation between living alone and social isolation among older adults, proactive measures are required to prevent and mitigate social isolation. This may entail community-based interventions, leveraging technology for social connections and fostering intergenerational interactions.

## 5 Conclusion

Our study provides compelling evidence that living arrangements significantly affect the mental health of Korean older adults, with social isolation and loneliness playing a critical role in the severity of depression. These findings highlight the protective effects of cohabitation and underscore the mental health risks associated with living alone, particularly in the context of South Korea’s rapidly aging population and the unique challenges posed by the COVID-19 pandemic. These insights are crucial for informing public health policies and interventions aimed at mitigating loneliness among older adults and improving their overall mental well-being.

However, this study had several limitations. The cross-sectional nature of the data limits our ability to infer causality between living arrangements and mental health outcomes. Additionally, the reliance on self-reported measures to assess mental health status introduces potential biases that could affect the accuracy of the findings. Furthermore, the study’s focus on South Korean older adults may limit the generalizability of the results to other cultural or geographical contexts, where different social dynamics may influence the relationship between living arrangements and mental health in various ways.

Future research should address these limitations by employing a longitudinal study design to better understand the causal relationships between living arrangements and mental health over time. Qualitative studies could also provide deeper insights into the experiences of social isolation and loneliness among older adults, offering a more nuanced understanding of how these factors affect mental well-being. Comparative studies across different cultural and geographical contexts could explore the universality of the findings and identify specific cultural or policy interventions that effectively address the challenges faced by older adults globally. Intervention studies are needed to test the effectiveness of strategies aimed at reducing loneliness and improving mental health among older adults living alone, including community-based programs, technology-driven social connectivity solutions, and housing policies promoting co-living arrangements.

Building on the findings of this study and addressing its limitations, future research can contribute to the development of more comprehensive and effective interventions to support the mental health of older adults. This is especially pertinent as societies worldwide grapple with the challenges of aging demographics and their implications for individual well-being, social cohesion, and public health infrastructure.

## Data availability statement

Publicly available datasets were analyzed in this study. This data can be found at: https://knhanes.kdca.go.kr/knhanes/sub03/sub03_02_05.do.

## Ethics statement

The studies involving humans were approved by the Institutional Review Board (2018-01-03-P-A and 2018-01-03-2C-A) at the Korea Disease Control and Prevention Agency. The studies were conducted in accordance with the local legislation and institutional requirements. The participants provided their written informed consent to participate in this study.

## Author contributions

GL: Conceptualization, Data curation, Formal analysis, Funding acquisition, Investigation, Methodology, Project administration, Resources, Software, Supervision, Validation, Visualization, Writing – original draft, Writing – review & editing. CK: Conceptualization, Data curation, Formal analysis, Funding acquisition, Investigation, Methodology, Project administration, Resources, Software, Supervision, Validation, Visualization, Writing – original draft, Writing – review & editing.
