# Supplementary material for: Social isolation and mental well-being among Korean older adults: a focus on living arrangements
Source: Front Public Health. 2024 Apr 24;12:1390459. doi: 10.3389/fpubh.2024.1390459 (PMC11076745; doi:10.3389/fpubh.2024.1390459)
Supplement: Supplementary file 1 [file Table_1.docx]

APPENDIX

Table 1. Vuong Test for Model Selection

| Model | Main Effect Model | | Main + Interaction Effect Model | |
| --- | --- | --- | --- | --- |
|  | ZIP | Poisson | ZIP | Poisson |
| AIC | 10,669.24 | 13,605.68 | 10,673.72 | 13,610.67 |
| BIC | 10,926.69 | 13,734.41 | 10,966.29 | 13,756.95 |
| Vuong test | ZIP model versus Poisson model | | ZIP model versus Poisson model | |
| Z-statistics* | 0.15** | ZIP = Poisson | 0.01** | ZIP = Poisson |
| N  (Zero observation) | 2,840 | 2,840 (1,376) | 2,840 | 2,840 (1,376) |

* Z= $\frac{\bar{L}}{S(L)}$ where $\bar{L}$ is the mean of the log-likelihood ratio differences beween the ZIP model and standard Poisson model across all observations. S(L) is the standard devidation of these log-likelihood ratio differences. If the Z statistic is greater than 1.96, then ZIP model will be preferred; if the Z-statitic is less than -1.96, the general Poisson model will be preferred; if the Z-statistic is between -1.96 and 1.96, then the both models are equally good.

** Not statistically significant at the level of 0.05

Table 2. Comparison of PHQ-9 Depression Severity Scores Between 2018 and 2020 Samples

|  | 2018 Sample | | | 2020 Sample | | |
| --- | --- | --- | --- | --- | --- | --- |
| PHQ-9 Score (Depression Severity) | Mean | SD | Frequency | Mean | SD | Frequency |
| 0 to 4  (None) | 0.87 | 1.26 | 1,234 | 0.91 | 1.26 | 1,131 |
| 5 to 9  (Mild) | 6.57 | 1.35 | 175 | 6.63 | 1.40 | 149 |
| 10 to 14  (Moderate) | 11.57 | 1.38 | 42 | 11.51 | 1.28 | 47 |
| 15 to 19  (Moderately Severe) | 16.46 | 1.26 | 28 | 16.58 | 1.72 | 12 |
| 20 to 27  (Severe) | 21.61 | 1.38 | 13 | 22.80 | 2.39 | 10 |

Table 3. Comparison of PHQ-9 Questionnaire Responses between 2018 and 2020 Samples

|  | 2018 Sample | | 2020 sample | |
| --- | --- | --- | --- | --- |
| Q1. Over the last 2 weeks….., little interest or pleasure in doing things | | | | |
| Category | Frequency | Percent | Frequency | Percent |
| 0 (Not at all) | 1,306 | 86.38 | 1,184 | 86.87 |
| 1 (Several days) | 97 | 6.42 | 101 | 7.41 |
| 2 (More than half the days) | 28 | 1.85 | 23 | 1.69 |
| 3 (Nearly every day) | 81 | 5.36 | 55 | 4.04 |
|  | Mean:0.26 SD: 0.74 | | Mean:0.23 SD: 0.67 | |
| Q2. Over the last 2 weeks….., feeling down, depressed, or hopeless | | | | |
| 0 (Not at all) | 1,315 | 86.97 | 1,198 | 87.77 |
| 1 (Several days) | 109 | 7.21 | 103 | 7.55 |
| 2 (More than half the days) | 24 | 1.59 | 25 | 1.83 |
| 3 (Nearly every day) | 64 | 4.23 | 39 | 2.86 |
|  | Mean:0.23 SD: 0.68 | | Mean:0.19 SD: 0.60 | |
| Q3. Over the last 2 weeks….., trouble failing or staying asleep, or sleeping too much | | | | |
| 0 (Not at all) | 1.053 | 69.78 | 935 | 68.40 |
| 1 (Several days) | 215 | 14.25 | 222 | 16.24 |
| 2 (More than half the days) | 53 | 3.51 | 56 | 4.10 |
| 3 (Nearly every day) | 188 | 12.46 | 154 | 11.27 |
|  | Mean:0.58 SD: 1.03 | | Mean:0.58 SD: 1.00 | |
| Q4. Over the last 2 weeks….., feeling tired or having little energy | | | | |
| 0 (Not at all) | 1,045 | 69.34 | 941 | 69.09 |
| 1 (Several days) | 259 | 17.19 | 248 | 18.21 |
| 2 (More than half the days) | 50 | 3.32 | 48 | 3.52 |
| 3 (Nearly every day) | 153 | 10.15 | 125 | 9.18 |
|  | Mean:0.54 SD: 0.96 | | Mean:0.52 SD: 0.93 | |
| Q5. Over the last 2 weeks….., poor appetite or overeating | | | | |
| 0 (Not at all) | 1,323 | 87.50 | 1,181 | 86.27 |
| 1 (Several days) | 95 | 6.28 | 96 | 7.01 |
| 2 (More than half the days) | 23 | 1.52 | 26 | 1.90 |
| 3 (Nearly every day) | 71 | 4.70 | 66 | 4.82 |
|  | Mean:0.23 SD: 0.70 | | Mean:0.25 SD: 0.72 | |
| Q6. Over the last 2 weeks….., feeling bad about yourself | | | | |
| 0 (Not at all) | 1,368 | 90.66 | 1,234 | 90.34 |
| 1 (Several days) | 85 | 5.63 | 82 | 6.00 |
| 2 (More than half the days) | 23 | 1.52 | 13 | 0.95 |
| 3 (Nearly every day) | 33 | 2.19 | 37 | 2.71 |
|  | Mean:0.15 SD: 0.53 | | Mean:0.16 SD: 0.56 | |
| Q7. Over the last 2 weeks….., trouble concentrating on things, such as reading the newspaper or watching television | | | | |
| 0 (Not at all) | 1,405 | 93.17 | 1,282 | 93.78 |
| 1 (Several days) | 47 | 3.12 | 48 | 3.51 |
| 2 (More than half the days) | 10 | 0.66 | 10 | 0.73 |
| 3 (Nearly every day) | 46 | 3.05 | 27 | 1.98 |
|  | Mean:0.13 SD: 0.56 | | Mean:0.10 SD: 0.48 | |
| Q8. Over the last 2 weeks….., moving or speaking so slowly that other people could have noticed? | | | | |
| 0 (Not at all) | 1,431 | 94.64 | 1,290 | 94.23 |
| 1 (Several days) | 44 | 2.91 | 48 | 3.51 |
| 2 (More than half the days) | 7 | 0.46 | 11 | 0.80 |
| 3 (Nearly every day) | 30 | 1.98 | 20 | 1.46 |
|  | Mean:0.10 SD: 0.46 | | Mean:0.09 SD: 0.43 | |
| Q9. Over the last 2 weeks…..,thoughts that you would be better off dead or of hurting yourself in some way | | | | |
| 0 (Not at all) | 1400 | 92.65 | 1,263 | 92.39 |
| 1 (Several days) | 68 | 4.50 | 74 | 5.41 |
| 2 (More than half the days) | 12 | 0.79 | 8 | 0.59 |
| 3 (Nearly every day) | 31 | 2.05 | 22 | 1.61 |
|  | Mean:0.12 SD: 0.49 | | Mean:0.11 SD: 0.46 | |
